# Preparation and Performance of PBAT/PLA/CaCO_3_ Composites via Solid-State Shear Milling Technology

**DOI:** 10.3390/polym16202942

**Published:** 2024-10-20

**Authors:** Xuehua Jia, Qilin Wen, Yanjun Sun, Yinghong Chen, Dali Gao, Yue Ru, Ning Chen

**Affiliations:** 1 State Key Laboratory of Polymer Materials Engineering, Polymer Research Institute of Sichuan University, Chengdu 610065, China; jiaxh@stu.scu.edu.cn (X.J.);; 2 Sinopec (Beijing) Research Institute of Chemical and Industry Co., Ltd., Beijing 100013, China

**Keywords:** poly(butylene adipate-co-terephthalate), poly(lactic acid), solid-state shear milling, mechanochemistry, biodegradable composite

## Abstract

Replacing traditional disposable, non-biodegradable plastic packaging with biodegradable plastic packaging is one of the key approaches to address the issue of “white pollution”. PBAT/PLA/inorganic filler composites are widely utilized as a biodegradable material, commonly employed in the field of packaging films. However, the poor dispersion of inorganic fillers in the polymer matrix and the limited compatibility between PBAT and PLA have led to inferior mechanical properties and elevated costs. In this work, we propose a simple and effective strategy to improve the dispersion of nano-CaCO_3_ in a PBAT/PLA matrix through solid-state shear- milling (S^3^M) technology, combined with mechanochemical modification and in situ compatibilization to enhance the compatibility between PBAT and PLA. The impact of varying milling conditions on the structure and performance of the PBAT/PLA/CaCO_3_ composites was investigated. During the milling process, PBAT and PLA undergo partial molecular chain fragmentation, generating more active functional groups. In the presence of the chain extender ADR during melt blending, more branched PBAT-g-PLA is formed, thereby enhancing matrix compatibility. The results indicate that the choice of milling materials significantly affects the structure and properties of the composite. The film obtained by milling only PBAT and CaCO_3_ exhibited the best performance, with its longitudinal tensile strength and fracture elongation reaching 22 MPa and 437%, respectively. This film holds great potential for application in the field of green packaging.

## 1. Introduction

Packaging serves as a crucial safeguard for the use, storage, and transportation of goods, constituting a necessity for human survival, livelihood, and production. Plastic packaging, particularly plastic film, is extensively utilized in numerous fields due to its ease of processing, low cost, lightweight, and superior performance, mainly including food packaging, pharmaceutical packaging, electronic product packaging, agricultural coverings, shopping bags, etc. [1]. Currently, about 430 million tons of plastic is produced yearly, according to the United Nations Environment Programme (UNEP), and approximately 36 percent of all plastic produced is for packaging. A total of 95% of the plastic used in packaging is disposed of after one use, as concludes a report by McKinsey [2]. However, owing to the challenges in recycling and the slow natural degradation, 85 percent of the plastic ends up in landfills or as hazardous waste, resulting in considerable resource wastage and severe environmental pollution. Meanwhile, biodegradable plastics, capable of decomposing entirely into CO_2,_ CH_4_, H_2_O, and inorganic salts, constitute significant environmentally friendly packaging alternatives and have been recognized as a key approach to address “white pollution”.

Poly(butylene adipate-co-terephthalate) (PBAT) is a commercial biodegradable polymer with properties similar to low-density polyethylene (LDPE), exhibiting high fracture elongation and good ductility, making it suitable for manufacturing films, such as shopping bags, garbage bags, mulch film, etc. [3,4,5,6]. However, the poor tensile strength of PBAT limits its applications. Polylactic acid (PLA) is another commercial biodegradable polymer derived from renewable biomass resources [7,8]. It is rigid and brittle, complementary to the performance of PBAT, and can effectively improve the problem of the low strength of PBAT. Researchers have conducted extensive studies on the structure and properties of PBAT/PLA blends [9,10], discovering that differences in the molecular chain structures between PBAT and PLA result in significant differences in solubility parameters, which subsequently lead to the issue of poor compatibility when simply mechanically blended [11,12]. Furthermore, PBAT/PLA blends are relatively expensive compared with traditional non-biodegradable plastics. In order to prepare biodegradable plastic films with high performance and low cost, PBAT is usually chosen as the matrix, PLA serves as a reinforcing agent, and a high content of fillers helps to reduce costs. Currently, widely used fillers include inorganic particles, such as CaCO_3_ [13], clays, montmorillonite [14], talc [15], and SiO_2_, and organic fillers, like thermoplastic starch [16,17] and lignin [18]. Among them, inorganic nanofillers are widely used as fillers for plastic due to their unique nanometer-size effects and low cost. However, the poor dispersion of nanofillers in PBAT/PLA matrixes often leads to aggregation, resulting in a decrease in mechanical properties. Therefore, surface modification and mechanical dispersion were developed to achieve the excellent dispersion of fillers [19,20]. Particularly, solid-state processing techniques, such as ball milling and solid-state shear milling, are capable of achieving good dispersion of fillers [21,22], with the polymer chain scission and grafting occurring under strong stress, leading to changes in the material structure and properties [23].

Highly efficient solid-state shear milling (S^3^M) processing is a unique processing technology developed by our team, featuring a unique three-dimensional shear structure, similar to a traditional Chinese stone mill. This structure is capable of providing a powerful compressive and shear force field, enabling a multitude of functions, such as crushing, dispersion, mixing, and activation. It can achieve the dispersion of inorganic micro–nanoparticles in a polymer matrix at room temperature, thereby enhancing the processing and mechanical properties of composites. The co-milling process can facilitate blending, compatibilization, and phase structure control between polymers with mismatched viscosities and incompatibilities. Previous research results have shown that this process can control the dispersed phase size of polymer blends at approximately 1 μm, significantly reduce aggregation, and achieve the uniform dispersion of nanofillers, resulting in effectively enhancing the performance of composites [24,25]. Wei employed S^3^M technology to recycle aluminum–plastic packaging waste. Following multiple milling cycles, the average particle size of the material was reduced from 700 μm to 226 μm, leading to a decrease in the phase domain size of the dispersed phase within the composite material. Consequently, this refinement significantly bolstered the tensile stress and impact strength properties of the material [26]. Additionally, the mechanical activation effect of the S^3^M process can effectively enhance the interfacial compatibility between domains. For example, Lai utilized the S^3^M technology for the recycling of waste acrylonitrile–-butadiene rubber (NBR)/poly(vinyl chloride) (PVC) insulation materials. The study revealed that during the milling process, the rubber molecular chains approached each other, leading to an enhancement in the interaction between polar groups and, consequently, a strengthened interaction between the polar rubber and plastic components. This phenomenon effectively improved the compatibility between NBR and PVC [27]. Furthermore, the S^3^M technology is relatively environmentally friendly due to its low-temperature requirements and the elimination of the need for initiators and solvents.

This study aimed to achieve the uniform dispersion of nano-CaCO_3_ in a PBAT/PLA matrix through S^3^M technology and improve the compatibility of PBAT and PLA through in situ compatibilization reactions combined with mechanochemical modification. The effects of different processing conditions on the structure and properties of PBAT/PLA/CaCO_3_ composites were investigated. It provides a new implementation strategy for the preparation of biodegradable packaging materials with high performance and low cost.

## 2. Materials and Methods

### 2.1. Materials

Poly(butylene adipate-co-terephthalate) (PBAT A400) was purchased from Zhuhai Kingfa Biodegradable Materials Co., Ltd. (Zhuhai, China) (T_g_ = −26.3 °C, T_m_ = 121.4 °C, and MI = 4.49 g/10 min (2.16 kg; 190 °C)). Polylactic acid (PLA 4032D) was purchased from Nature Works^®^ LLC (Minneapolis, MN, USA) (T_g_ = 63.2 °C; T_m_ = 168.9 °C). Nanometer-activated calcium carbonate (nano-CaCO_3_; CCR-800) was purchased from Foshan Biaorui Chemical Co., Ltd. (Foshan, China). The chain extender (BASF ADR4370-F) was purchased from Dongguan Yingsheng Plastic Chemical Co., Ltd. (Dongguan, China).

### 2.2. Preparation of PBAT/PLA/CaCO_3_ Composites

First, PBAT, PLA, and CaCO_3_ were dried at 80 °C for 6 h to remove moisture. All formulations were prepared with a ratio of 70% polymer (where PBAT–:PLA = 7:3), 30% CaCO_3_, and an additional 0.45% ADR. Then, a portion of the raw materials was manually pre-mixed according to the ratios (as shown in step 1 in Table 1) and added to the S^3^M equipment for milling. The milling parameters were set as follows: a rotation speed of 50 rpm, a pressure of 2–3 MPa, and milling performed twice. Temperature control of the milling disc was achieved by circulating cooling water at a constant temperature inside the disc.

The mixture obtained from the S^3^M equipment (processing step 1) was then extruded together with other components (processing step 2) by a twin screw. The temperatures in different zones were set at 150 °C, 165 °C, 170 °C, 175 °C, and 175 °C, with the die temperature at 160 °C and a rotation speed of 50 rpm. The obtained filament strips were pelletized, dried at 80 °C for 6 h, and then hot-pressed into 0.5 mm thick sheets for testing.

**Figure 1:**
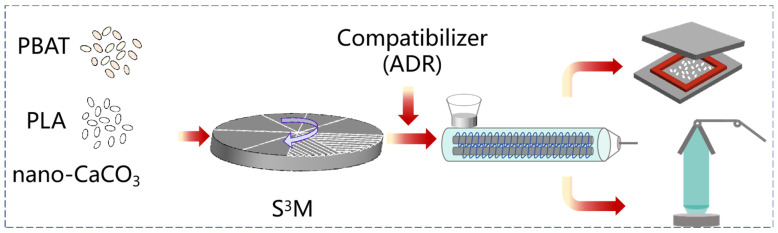
Processing of PBAT/PLA/CaCO_3_ composites.

The preparation of PBAT/PLA/CaCO_3_ films was as follows: The composite with the best performance was selected for film blowing and tested for its mechanical properties. The temperature of each zone during film blowing was 150 °C, 165 °C, 175 °C, 168 °C, and 155 °C. The general processing flow is illustrated in Figure 1.

## 3. Characterization

### 3.1. Gel Permeation Chromatography (GPC)

The molecular weights of PBAT, PLA, and the PBAT/PLA blend were determined by GPC (Agilent-1260, Refractive Index detection), using chloroform as the eluent (with a flow rate of 1 mL/min) to maintain good solubility at 35 °C. Polystyrene (PS) standards were utilized for calibration to determine the average molecular weight (Mw) and polydispersity index (PDI), aiming to investigate the impact of solid-state shear milling on the molecular chains of the materials. The test sample was obtained by dissolving the composite material in chloroform, followed by centrifugation to remove the insoluble CaCO_3_ component.

### 3.2. Fourier- Transform Infrared Spectra (FTIR)

Fourier-transform infrared spectroscopy (Nicolet 6700 spectrometer, Thermo Fisher Scientific Inc., Waltham, MA, USA) was used to study the interactions between PLA, PBAT, ADR, and their mixtures over a scanning range of 4000–650 cm^−1^, with a resolution of 4 cm^−1^ and 32 scans.

### 3.3. Scanning Electron Microscopy (SEM)

The brittle fracture surface and tensile fracture surface of the PBAT/PLA/CaCO_3_ composites were observed by a scanning electron microscope (Apreo S HiVoc, FEI Instrument Co., Ltd., Hillsboro, OR, USA) at an accelerating voltage of 20 kV. The composites were pre-treated with low-temperature brittle fracture in liquid nitrogen, followed by gold sputtering on the sample surface to observe the fracture morphology. The distribution of specific elements in the samples was observed by energy-dispersive X-ray spectrometry (Octane Elect Super, EDAX, Pleasanton, CA, USA) connected to the scanning electron microscope.

### 3.4. Rheological Behavior

The rheological properties of the PBAT/PLA/CaCO_3_ composites at 175 °C were studied using a rotational rheometer (TA R2000, TA Instrument, Co., Ltd., New Castle, DE, USA). The samples were placed on parallel plates with a diameter of 25 mm and tested with a 1 mm gap. The dynamic frequency sweep ranged from 0.05 to 100 rad/s, with a strain set at 0.1% in the linear viscoelastic region.

### 3.5. Mechanical Performance Testing

The mechanical properties of the composites and films were conducted according to ISO 527 standards using an Instron material-testing machine (Instron 5567, Instron Co., Ltd. Norwood, MA, USA). The composite sheet was cut into a dumbbell shape, and the tensile rate was set at 50 mm/min. The sample thickness was measured using a micrometer screw gauge. The tensile strength (TS) and elongation at break (EB) of the samples were recorded. At least five dumbbell specimens were cut from each sample for testing, and the average values were taken after testing. Dynamic mechanical analysis (DMA) was performed using a DMA TQ850 (TA Instrument, Co., Ltd., New Castle, DE, USA), providing the storage modulus (E’) and loss factor (tanδ) as a function of temperature. The sample dimensions were 20 × 4 × 0.5 mm^3^. Scanning was performed in tensile mode with a heating rate of 3 °C/min, a temperature range of −60 °C to 130 °C, a displacement amplitude of 20 μm, and a frequency of 1 Hz.

### 3.6. Thermal Properties

Differential scanning calorimetry (DSC) was measured using a Q20 instrument (TA Instrument, Co., Ltd., New Castle, DE, USA). The sample weight ranged from 5 to 10 mg. The heating and cooling processes were divided into three steps. Firstly, each sample was heated from −60 °C to 200 °C and maintained in a molten state for 3 min (first heating) to eliminate thermal history. Subsequently, the sample was cooled to −60 °C, followed by reheating to 200 °C (second heating). Both heating and cooling rates were set at 10 °C/min. The glass transition temperature (T_g_), melting temperature (T_m_), and cold crystallization temperature (T_cc_) were determined from the results obtained through heating and cooling cycles. The crystallinity of PLA was calculated using Equation (1):

(1) $$\chi_{PLA}=\frac{∆H_{m}-∆H_{cc}}{∆H_{m}^{0}\times W_{PLA}}$$

where $∆H_{m}$ is the sample’s melting enthalpy, $∆H_{cc}$ is the cold crystallization enthalpy of PLA, $W_{PLA}$ represents the weight fraction of PLA, and $∆H_{m}^{0}$ is the melting enthalpy of 100% crystalline PLA (93.6 J/g).

Thermogravimetric analysis of the composites was carried out with a Q50 thermal gravimetric analyzer (TA Instrument, Co., Ltd., New Castle, DE, USA). Each sample was heated from room temperature to 650 °C at a rate of 10 °C/min in a nitrogen environment.

## 4. Results and Discussion

### 4.1. Structural Changes in Composites

**Figure 2:**
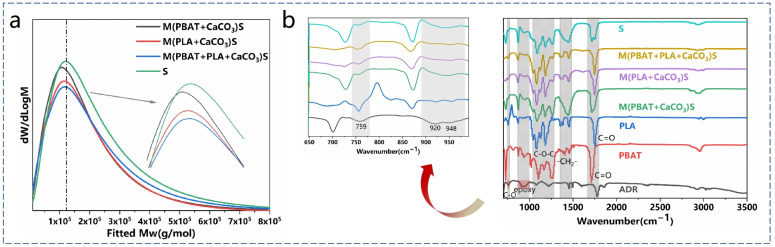
(**a**) GPC traces of PBAT, PLA, and PBAT/PLA blends; (**b**) FTIR spectra of the composites with different milling conditions.

The impact of S^3^M on the molecular weight of the blend was investigated using GPC, as depicted in Figure 2a, confirming that the milling process led to the breakage of some molecular chains, resulting in a decrease in the molecular weight of the PBAT/PLA blend. Particularly, after milling all materials together, the molecular weight decreased from 136,389 to 106,939 (as shown in Table 2). To further investigate the impact of S^3^M on the molecular weight of the material, we roughly estimated the weight-average molecular weight (Mw) of the composites based on the corresponding proportions of their constituents. When no milling was applied (S), the calculated Mw of the composite was approximately 134,000, which was relatively close to the result obtained through GPC. This suggests that the primary reduction in molecular weight occurred during the milling stage. Based on this understanding, we calculated that after milling, the Mw of PBAT decreased from 120,000 to approximately 88,000 (M(PBAT + CaCO_3_)S), and the Mw of PLA decreased from 168,000 to approximately 99,000 (M(PLA + CaCO_3_)S). Therefore, during milling, the relatively unstable ester bonds were more prone to breakage, forming end-group functionalities. Based on previous reports, during the subsequent melt mixing process, these end-group functional groups were prone to react with the epoxy groups of ADR, potentially leading to the formation of graft copolymers such as PBAT-g-PLA [28]. Similar reactions have been reported upon the addition of POSS containing a large number of epoxy functional groups [29].

To further analyze the impact of S^3^M on the structure of the composites, FTIR was conducted on the composites, with the results shown in Figure 2b. The two peaks near 1711 cm^−1^ and 1250 cm^−1^ correspond to the carbonyl stretching vibration peak and the C-O stretching vibration peak of PBAT, respectively. The peak at 727 cm^−1^ corresponds to the out-of-plane bending vibration of C-H on a para-disubstituted benzene ring, while the peaks within the range of 800–950 cm^−1^ correspond to the out-of-plane bending vibration peaks of C-H in the benzene ring. The peak at 1750 cm^−1^ corresponds to the carbonyl stretching vibration peak of PLA, while the characteristic ester C-O stretching vibrations of PLA appear at 1042 cm^−1^, 1080 cm^−1^, 1129 cm^−1^, and 1180 cm^−1^ [29,30]. The epoxy band of ADR and the C-O stretching vibration peak within the epoxy are [28,29] located at 920 cm^−1^ and 759 cm^−1^, respectively [31]. In the graph, it is observed that the peak at 920 cm^−1^ in the composite material disappears, which is attributed to the reaction involving the chain extender ADR. The carboxyl and hydroxyl end groups of PLA and PBAT are prone to react with the epoxy functional groups of ADR, leading to the formation of grafts that are often located at the interface between the two phases, thereby enhancing compatibility [28,32,33].

### 4.2. Microscopic Morphologies of Composites

**Figure 3:**
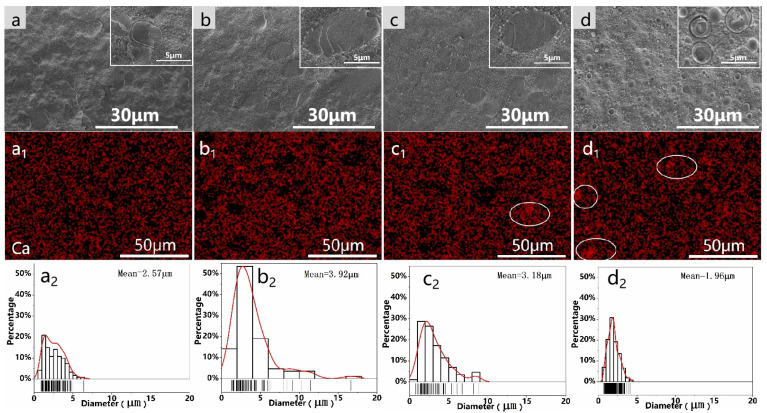
SEM images of the fractured cross-sections for (**a**) M(PBAT + CaCO_3_)S, (**b**) M(PLA + CaCO_3_)S, (**c**) M(PBAT + PLA + CaCO_3_)S, and (**d**) S; EDS mapping for (**a_1_**) M(PBAT + CaCO_3_)S, (**b_1_**) M(PLA + CaCO_3_)S, (**c_1_**) M(PBAT + PLA + CaCO_3_)S, and (**d_1_**) S; size distribution of the dispersed phase for (**a_2_**) M(PBAT + CaCO_3_)S, (**b_2_**) M(PLA + CaCO_3_)S, (**c_2_**) M(PBAT + PLA + CaCO_3_)S, and (**d_2_**) S. (The density of the lines in the bottom bar chart reflects the distribution of the dispersed phase in the corresponding sizes within the statistical sample).

As shown in Figure 3, in order to understand the microstructure of the composites, the cross-sections were characterized using SEM, and the size of the dispersed phase was statistically analyzed. Based on their cross-sectional morphology (a–d), the composites exhibited a distinct island structure, with PLA dispersed in PBAT in circular and elliptical shapes. Upon the incorporation of PLA during the milling process, the size of the dispersed phase gradually increased, with the morphology progressively evolving toward an elliptical and elongated shape. As observed in Figure 3(a_2_–d_2_), the average size of the dispersed phase in the composite without milling was 1.96 μm. However, after milling, the average size of the dispersed phase increased, particularly when PLA was involved in the milling process. Compared with direct melt blending, as shown in (d), S^3^M (a–c) led to a gradual blurring of the two-phase interface between PBAT and PLA, especially when only PBAT and CaCO_3_ were milled. This indicates that S^3^M enhances the interfacial adhesion between the two phases and the compatibility between the resins. This enhancement may be attributed to the strong three-dimensional shear forces during milling, leading to chain breakage and the formation of more end-group functionalities, facilitating the formation of graft copolymers at the interface during milling and subsequent melt blending processes to improve compatibility.

To further analyze the dispersion of CaCO_3_ in the PBAT/PLA matrix, energy-dispersive X-ray spectroscopy (EDS) was used to analyze the fractured surfaces of the materials. In Figure 3(a_1_–d_1_), it can be observed that after milling, the dispersion of Ca elements improved, and the aggregation of CaCO_3_ particles decreased, indicating that S^3^M technology can effectively enhance filler dispersion. Therefore, there is a potential to increase the filler content without compromising the overall mechanical properties, thereby further reducing costs.

### 4.3. Rheological Behavior of Composites

**Figure 4:**
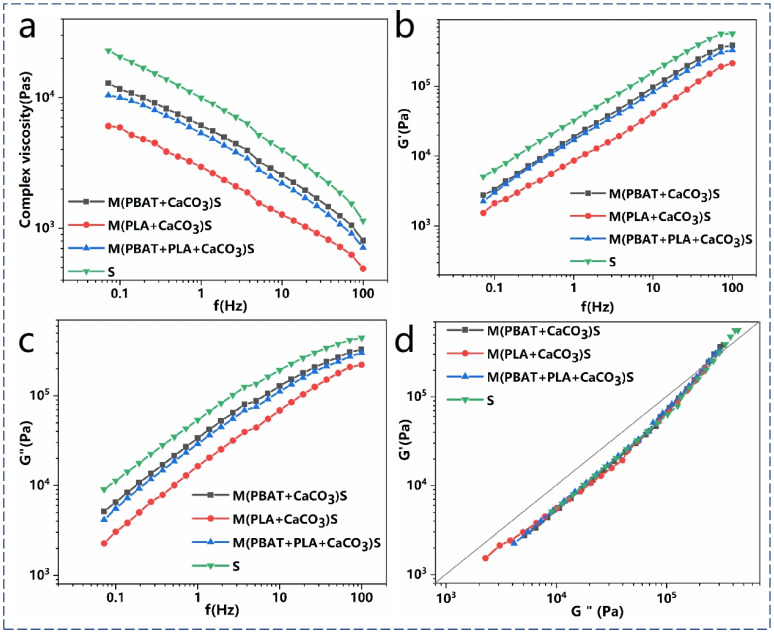
(**a**) Complex viscosity, (**b**) storage modulus, (**c**) loss modulus, and (**d**) Han curves of the PBAT/PLA/CaCO_3_ composites with different milling conditions.

In an extruded blown film of biodegradable polyester, there is a high demand for melt strength. Materials with low melt strength can lead to various processing anomalies, such as a narrow processing window, unstable film bubbles, and inconsistent extrusion rates. The rheological properties of the PBAT/PLA/CaCO_3_ composites were studied in the linear viscoelastic region in order to provide some key information for processing. Figure 4 illustrates the relationship between the complex viscosity, storage modulus, loss modulus, and oscillation frequency of the PBAT/PLA/CaCO_3_ composites measured at 175 °C. A higher storage modulus and appropriate complex viscosity ensure melt strength and flowability during processing [13]. Additionally, the melt rheological properties of the composite material are directly related to the dispersion of fillers in the matrix, as filler aggregation in the matrix increases complex viscosity [34]. The results show significant shear thinning behavior in the composite material, primarily due to the disruption of molecular structures by shear forces, leading to viscosity reduction.

S^3^M technology reduces the viscosity and modulus of the composite material, mainly because the strong three-dimensional shear forces during milling cause a certain degree of chain breakage, reducing the molecular weight and weakening intermolecular entanglement. Particularly when PLA is involved in milling, as PLA is an aliphatic polyester that is rigid, brittle, and relatively heat-sensitive, it is more prone to chain breakage during milling. Additionally, milling improves the dispersion of CaCO_3_ in the matrix, further reducing the viscosity of composites.

Both the storage modulus and loss modulus increase with the angular frequency, consistent with linear viscoelastic theory. The increase in the energy storage modulus with increasing frequency is mainly due to the fact that the de-entanglement rate between CaCO_3_ and the molecular chains of the composite material cannot keep up with the deformation rate after the composite material has been subjected to force, and, thus, more energy is required to disrupt the entanglement deformation of the molecular chains. With increasing shear frequency, the friction between the composite material molecular chains and between the molecular chains and CaCO_3_ increases, leading to more energy dissipation during deformation, resulting in an increase in the loss modulus with frequency.

In multiphase polymer systems, the heterogeneity causes the rheological behavior of the system to exhibit different viscoelastic responses at different shear frequencies, making the Han plot useful for studying the compatibility of multiphase polymer blends. Figure 4d shows the relationship curve between G′ and G″ of the blend, indicating a nonlinear relationship within the tested frequency range, suggesting system incompatibility. The decrease in the storage modulus and loss modulus at the end of the curve may be attributed to the solid-state shear milling partially reducing intermolecular entanglement.

### 4.4. Mechanical Properties of Composite Materials

**Figure 5:**
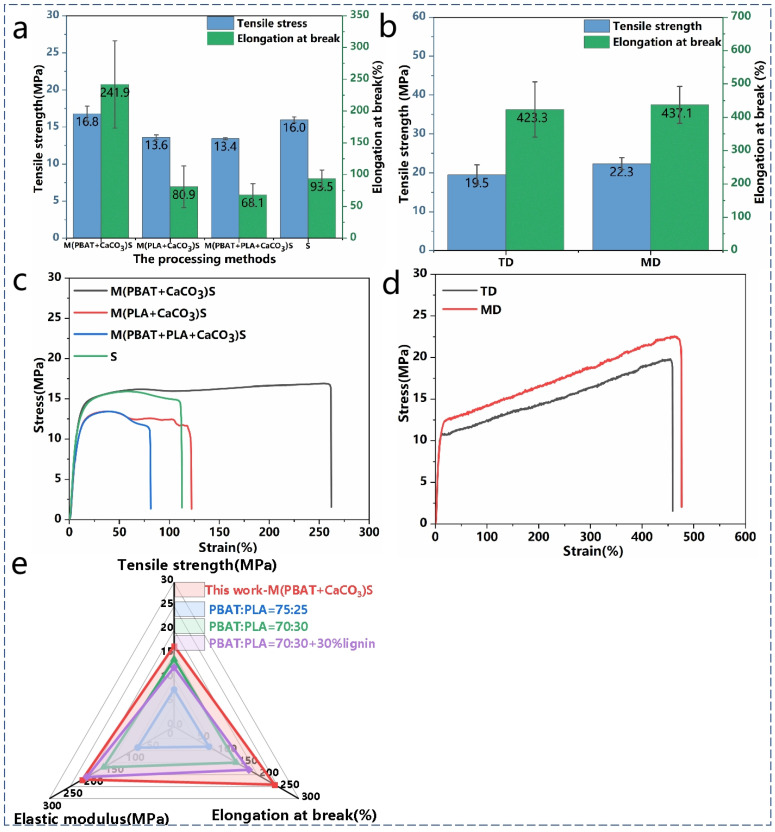
(**a**,**c**) Tensile properties of composites with different milling conditions; (**b**,**d**) tensile properties of blown film samples for M(PBAT + CaCO_3_)S. (**e**) radar chart for comparison of tensile properties [36,37,38].

The mechanical properties of the composites were tested using a tensile testing machine, and the relevant data are presented in Table 3. As shown in Figure 5a,c, the S^3^M technology and milling conditions had a significant impact on the mechanical properties of the composites, particularly on the fracture elongation. Composites obtained by milling PBAT and CaCO_3_ only exhibited higher tensile strength and fracture elongation, mainly because CaCO_3_ was more dispersed in the PBAT matrix, achieving a uniform dispersion of the inorganic powder in the resin matrix. Furthermore, after milling, PBAT experienced chain breakage, leading to viscosity reduction and a decrease in the viscosity difference with PLA, improving their compatibility and stress transfer during tension.

Compared with PBAT, PLA is more sensitive to temperature, and the heat and shear forces generated during milling can cause a certain degree of degradation in PLA, resulting in a decrease in the tensile strength of the composite. Additionally, as PLA gradually takes on an elongated form with larger dimensions during milling, which is not conducive to stress transfer, brittle fractures occur, leading to a decrease in fracture elongation. Figure 5e presents a radar chart comparing the tensile properties of the M(PBAT + CaCO_3_)S sample from this experiment with PBAT/PLA materials reported in other literature, indicating the good tensile performance of the material.

As shown in Figure 5b,d, the material milled with only PBAT and CaCO_3_ was used for film blowing and tested for its tensile properties. The results show that the tensile strength of the material was more than 19 MPa, and the elongation at break was as high as 420%, which has a large application prospect in the field of green packaging.

**Figure 6:**
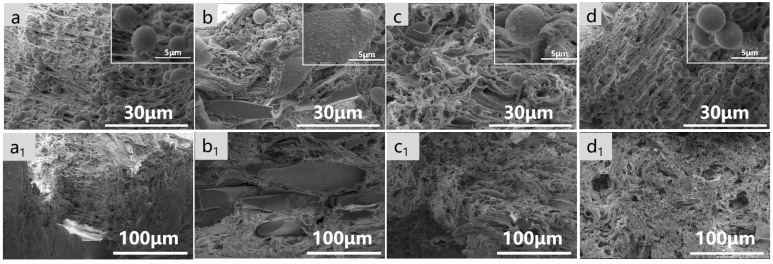
SEM images of the tensile sections for (**a**,**a_1_**) M(PBAT + CaCO_3_)S, (**b**,**b_1_**) M(PLA + CaCO_3_)S, (**c**,**c_1_**) M(PBAT + PLA + CaCO_3_)S, and (**d**,**d_1_**) S.

To further understand the stress deformation behavior of the materials, the tensile fracture surfaces were observed using SEM, as shown in Figure 6. The results reveal that the relatively rigid PLA was dispersed in PBAT, resembling an elastomer, in a spherical form. The fracture surface was rough, with numerous fibrous whiskers and long, tough bands. In composites that were not milled but directly melt-blended, CaCO_3_ was primarily dispersed in PBAT. With the involvement of PLA in milling, the amount of CaCO_3_ on the surface of PLA gradually increased. Additionally, it can be observed in the images that after PLA participated in milling, there were more elongated dispersed phases present, especially in materials where only PLA and CaCO_3_ were milled, leading to some brittle fractures. The factors influencing the breakage of dispersed-phase particles included external and internal forces. It is possible that when PLA is involved in milling, the dispersed phase changes from a granular to a powdery and elongated form, resulting in a decrease in the dispersed phase size and deformation stress. Furthermore, during milling, the breaking of PLA molecular chains reduces the melt viscosity and elastic forces, decreasing the internal forces that resist breakage. Therefore, according to the droplet dispersion mechanism, the deformation of the dispersed particles is not enough to break up when they rotate in the direction parallel to the shear force, making it difficult for further breakage to occur [35]. Due to the presence of rigid PLA particles, when the material is subjected to tensile orientation, external forces lead to the appearance of numerous cracks in the material. As the tensile process continues, these cracks propagate and eventually lead to fracture. Larger dispersed phase sizes are not conducive to stress transfer.

**Figure 7:**
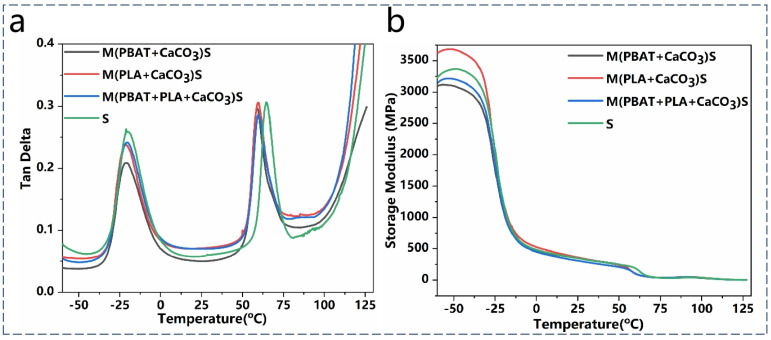
(**a**) Tan delta, (**b**) storage moduli of the blends.

To further investigate the effect of S^3^M on the compatibility of PBAT and PLA, the thermomechanical behavior of the composites was analyzed using dynamic mechanical analysis (DMA). Figure 7a,b show the relationships between the tanδ, storage modulus, and temperature for the composites. Table 4 presents the glass transition temperatures of PLA and PBAT, measured by DMA. It can be observed that the T_g_ of PBAT and PLA appeared at around −21 °C and 60 °C, respectively, indicating the immiscibility of the system and the presence of phase separation.

Compared with direct melt blending, S^3^M resulted in a decrease in the T_g_ of both PBAT and PLA. This was mainly due to molecular chain breakage and a reduction in molecular weight and entanglement between chains during the milling process. However, the difference in the T_g_ of PBAT and PLA decreased, indicating an improvement in the compatibility between the two phases. Particularly, when only PBAT and CaCO_3_ were milled, the T_g_ difference decreased by 4.81 °C compared with direct blending, suggesting better compatibility in the composites. This improvement in compatibility corresponds to the good mechanical performance of the films observed in the tensile tests.

In Figure 7b, a sharp decrease in the storage modulus is observed in the ranges of −35 to −20 °C and 50 to 65 °C, corresponding to the glass transition behaviors of PBAT and PLA, respectively.

### 4.5. Thermal Properties of Composites

**Figure 8:**
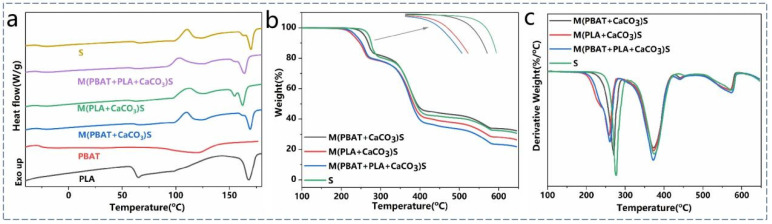
(**a**) DSC, (**b**) TGA, and (**c**) DTG curves of the composites with different milling conditions.

The thermal properties of pure PBAT, PLA, and the PBAT/PLA/CaCO_3_ composites were investigated using DSC. The second heating curves are shown in Figure 8a, and Table 5 provides the corresponding temperatures, enthalpies, and the crystallinity of PLA. The T_g_ of PBAT and PLA in the blends appeared at around −26 °C and 62 °C, respectively, indicating the immiscibility of the system. The materials treated with S^3^M corresponded to a decrease in the T_g_ and melting point of PLA, especially when only PLA and CaCO_3_ were milled, which is consistent with the DMA result. This may be attributed to the brittleness and sensitivity of PLA to shear forces and heat during the milling process, leading to easier chain breakage, reduced molecular weight, decreased chain entanglement, and increased free volume.

In the heating curves of each blend, cold crystallization peaks and double melting peaks of PLA were observed. Due to the characteristics of PLA, including a long crystallization induction period, slow crystallization rate, and imperfect crystallization, PLA produced through conventional processing methods involving rapid cooling from the melt typically exhibits low crystallinity or an amorphous state. When the temperature of amorphous PLA rises between T_g_ and T_m_, ordered crystalline structures form through the local adjustment and refinement of the molecular chains. The double melting peaks result from the unstable α′ crystals formed during cold crystallization in PLA, exhibiting a phenomenon of melt recrystallization and remelting during heating [39].

After PBAT was involved in milling, the cold crystallization temperature of the material decreased, particularly in M(PBAT + PLA + CaCO_3_)S, where the Tcc decreased by 7.8 °C. This could be because, after milling, the relatively lower molecular weight of PLA makes it easier to crystallize. Additionally, PBAT acts as a heterogeneous nucleating agent for PLA crystallization. Therefore, when PBAT is milled into a powdered form, the smaller particle size enhances nucleation effects. The crystallinity of PLA in the composite materials was relatively low, primarily due to the lack of orientation stretching. Processing techniques such as blow molding or biaxial stretching in later stages can effectively increase the crystallinity.

The thermal performance of the samples was evaluated using a thermogravimetric analyzer, and the TGA and DTG curves are shown in Figure 8b,c. The relevant temperatures are listed in Table 6. The degradation process of all blends consisted of three steps: the degradation of PLA and PBAT and the decomposition of CaCO_3_ into carbon dioxide and calcium oxide. The presence of longer molecular chain segments and aromatic structures in PBAT resulted in a higher thermal decomposition temperature compared with PLA. The research conducted by Pablos et al. indicates that the presence of metal ions such as Ca^2+^ in the filler CaCO_3_ may catalyze the thermal degradation of polymers [40].

From the thermogravimetric curves, it is evident that milling led to a decrease in the initial decomposition temperature of the materials, indicating a reduction in thermal stability, possibly due to chain breakage and a decrease in molecular weight during the milling process. The DTG curves show that milling primarily affected the thermal performance of PLA. Compared with the un-milled materials, the materials involving milled PLA exhibited reductions of 16.2 °C and 19.0 °C in the T_max-PLA_. This could be attributed to PLA being an aliphatic polyester, with its molecular chains being more sensitive to shear forces and heat during milling, making them more prone to breakage, while PBAT with rigid benzene rings demonstrates better thermal stability. Therefore, S^3^M primarily impacts the thermal stability of PLA but does not affect processing and usage.

## 5. Conclusions

This study utilized S^3^M to enhance the dispersion of nano-CaCO_3_ fillers in a PBAT/PLA matrix. Additionally, by incorporating ADR as a compatibilizer, a combination of in situ compatibilization and mechanochemistry compatibilization was employed to improve the compatibility between PBAT and PLA. This study investigated the effects of the milling conditions on the structure and properties of PBAT/PLA/CaCO_3_ composites.

The results demonstrate that S^3^M improved the compatibility between PBAT and PLA, further promoting the uniform dispersion of CaCO_3_ in the matrix. However, the thermal stability of the materials decreased due to chain breakage and partial degradation during the milling process. The milling conditions significantly influenced the final structure and properties of the composites. Consequently, in the subsequent experiments, the focus was solely on the milling of PBAT and CaCO_3_, aiming to achieve a homogeneous dispersion of CaCO_3_ within the PBAT matrix. Tensile tests revealed that following the film-blowing process, the longitudinal tensile strength and elongation at the break of the resulting film reached 22 MPa and 437%, respectively, showcasing significant potential for applications in the realm of sustainable packaging.

## Figures and Tables

**Table 1:** Experimental formulations and processing flow.

Component Content	Sample Code	Processing Step 1: S^3^M Conditions	Processing Step 2: Twin-Screw Blending Conditions
PBAT–:PLA = 7:3 (70%)	M(PBAT + CaCO_3_)S	PBAT + CaCO_3_	Step 1 + PLA + ADR
ADR = 0.45%	M(PLA + CaCO_3_)S	PLA + CaCO_3_	Step 1 + PBAT + ADR
CaCO3 = 30%	M(PBAT + PLA + CaCO_3_)S	PBAT + PLA + CaCO_3_	Step 1 + ADR
	S	-	PBAT + PLA + CaCO_3_ + ADR

**Table 2:** The molecular weights and distributions of PBAT, PLA, and PBAT/PLA blends.

Samples	M_w_ (g/mol)	PD
PBAT	119,900	2.23
PLA	168,310	2.21
M(PBAT + CaCO_3_)S	112,393	2.27
M(PLA + CaCO_3_)S	113,688	3.91
M(PBAT + PLA + CaCO_3_)S	106,963	3.44
S	136,389	2.20

**Table 3:** Tensile strength and elongation at break of composites.

Sample	Tensile Strength (MPa)	Elongation at Break (%)
M(PBAT + CaCO_3_)S	16.8 ± 1.0	241.9 ± 68.8
M(PLA + CaCO_3_)S	13.6 ± 0.3	80.9 ± 32.8
M(PBAT + PLA + CaCO_3_)S	13.4 ± 0.1	68.1 ± 17.8
S	16.0 ± 0.4	93.5 ± 13.8

**Table 4:** Glass transition temperatures by DMA test.

Sample	T_g(PBAT)_/°C	T_g(PLA)_/°C	△T_g_/°C
M(PBAT + CaCO_3_)S	−21.1	58.9	80.0
M(PLA + CaCO_3_)S	−21.6	59.6	81.2
M(PBAT + PLA + CaCO_3_)S	−20.5	59.9	80.4
S	−20.1	64.7	84.8

**Table 5:** Some thermal performance parameters of materials tested by DSC.

Sample	T_g(PBAT)_ (°C)	T_g(PLA)_ (°C)	T_cc_ (°C)	∆H_cc_ (J/g)	T_m_ (°C)	∆H_m_ (J/g)	X_c(PLA)_ (%)
PBAT	−26.3	-	-	-	121.4	17.0	-
PLA	-	62.8	-	-	168.3	14.0	14.9
M(PBAT + CaCO_3_)S	−26.6	62.4	108.9	6.1	169.3	7.0	4.4
M(PLA + CaCO_3_)S	−27.6	59.2	109.4	7.8	162.5	8.6	4.0
M(PBAT + PLA + CaCO_3_)S	−27.7	60.1	102.0	6.4	164.2	7.8	7.3
S	−27.0	62.7	109.7	6.8	170.0	7.7	4.6

**Table 6:** Thermal weight loss temperature of materials tested by TGA.

Samples	T_5%_ (°C)	T_max-PLA_ (°C)	T_max-PBAT_ (°C)
M(PBAT + CaCO_3_)S	261.1	270.4	373.7
M(PLA + CaCO_3_)S	230.9	257.8	373.9
M(PBAT + PLA + CaCO_3_)S	237.3	260.5	372.7
S	269.5	276.8	374.5

## Data Availability

The authors confirm that the data supporting the findings of this study are available within the article.
